# Physical aspects of electro osmotically interactive Cilia propulsion on symmetric plus asymmetric conduit flow of couple stress fluid with thermal radiation and heat transfer

**DOI:** 10.1038/s41598-023-45595-1

**Published:** 2023-10-28

**Authors:** Noreen Sher Akbar, Taseer Muhammad

**Affiliations:** 1 DBS&H, CEME, National University of Sciences and Technology, Islamabad, Pakistan; 2 Department of Mathematics, College of Science, King Khalid University, 61413 Abha, Saudi Arabia

**Keywords:** Biophysics, Mathematics and computing

## Abstract

A novel mathematical analysis is established that summits the key features of Cilia propulsion for a non-Newtonian Couple Stress fluid with the electroosmosis and heat transfer. In such physiological models, the conduit may have a symmetric or asymmetric configuration in accordance with the biological problem. Being mindful of this fact, we have disclosed an integrated analysis on symmetric in addition to asymmetric conduits that incorporates major physiological applications. The creeping flow inference is reviewed to model this realistic problem and exact solutions are computed for both the conduit cases. Graphical illustrations are unveiled to highlight the physical aspects of cilia propulsion on symmetric in addition to asymmetric conduit and an inclusive comparison study is conveyed. The flow profile attains higher values for an asymmetric conduit in relation to the symmetric. Likewise, the pressure rise and pressure gradient also score high for asymmetric conduit in relation to the symmetric conduit. A visual representation of flow inside symmetric as well as asymmetric conduit is provided by streamline graphs and temperature profile as well.

## Introduction

The foremost physiological flow phenomenon that emerges in living beings incorporate the functionality of cilia that are micro structures available in almost each cell of body and their consistent operation follows a travelling wave parallel to the conduits wall. This subsequently leads to the propulsion of biological fluids in many of the physiological problems such as water propulsion in various cells, mucus propulsion in respiratory tract, function of fallopian tubes, function of reproductive tracts etc. Lardner and Shack^1^ had discussed the details of cilia transportation in numerous biological models. Satir^2^ had scrutinized an intrigued model on cilia movements. Liron^3^ had unveiled the mathematical assessment on fluid propulsion due to metachronal effects of cilia between two aligned plates. Sleigh et al.^4^ had modeled the mucus transportation problem by consistent operations of cilia. Satir and Sleigh^5^ had featured the role of cilia in respiratory system with mucus propulsion. Gauger et al.^6^ had incorporated the concept of artificial cilia to analyze the flow characteristics at a negligible Reynolds number with MHD effects. Many researchers have formulated novel mathematical models that incorporate cilia driven flow^7–12^.

Some of the certain physiological flow problems incorporate the integrated elucidation of cilia propelled flow alongside Peristaltic flow mechanism. Peristalsis is the process of fluid transmission inside a conduit due to sinusoidal wall fluctuations. Barton and Raynor^13^ had scrutinized the flow inside a tube by utilizing long wavelength inference with sinusoidal wall fluctuations. Siddiqui and Schwarz^14^ had revealed the composite analysis on pressure driven flows and peristaltic flow of non-Newtonian fluids. Tripathi^15^ had mathematically handled a realistic model of chyme flow due to sinusoidal fluctuations of intestine walls. Tripathi and Beg^16^ had featured the drug delivery applications of Peristalsis via nanofluids. Akbar et al.^17^ had reviewed the prime features of peristaltic flow for an asymmetric conduit under MHD effects. Akbar and Khan^18^ had elaborated an innovative model of cilia governed flow inside a conduit having sinusoidal wall movements. Akbar^19^ had published the mathematical model of cilia propelled flow of nanofluids inside a conduit with sinusoidal walls. Ashraf et al.^20^ had considered the cilia propelled flow inside a fallopian tube with non-Newtonian fluid model.

Electro-osmotic flow is a crucial factor in various micro-channel processes and plays a significant role in biotechnology applications where there is an inherent charge imbalance. Notable applications of this phenomenon include tissue culture, cell scaffolding systems, pharmacodynamics, and medical devices at the nanoscale^21^. Tripathi et al.^22^ conducted a study on the electro-thermal peristaltic transport of nanofluid in a finite microchannel, incorporating the Chakraborty-Roy nanofluid electrokinetic formulation. Ijaz et al.^23^ investigated the impact of electro-osmosis on bio-nanofluid containing non-spherical particles within a curved channel. Their computational results showed that the introduction of blade-shaped particles led to an increase in heat transfer. In a recent study, Khan et al.^24^ explored how radiation influences electro-osmosis modulated peristaltic flow within a tapered channel, using Prandtl nanofluid. They discovered that isothermal lines expanded with an increase in the electro-osmotic parameter.

Recent research has placed a greater emphasis on studying thermal convective flows to understand how thermal energy transfers from one region to another in various conductive and convective processes. The rate of thermal energy transfer is influenced by temperature variations within the region of interest. However, when it comes to thermal radiation, energy transfer between two bodies depends on the absolute temperature difference. Thermal radiation finds numerous applications in the field of biomedicine. Due to its relevance in biomedicine and medical treatments, the study of the impact of thermal radiation with double diffusion has become a significant research area. Infrared radiation (IR) is a commonly used technique for applying heat treatment to different parts of the human body. Infrared radiation consists of electromagnetic waves that fall within the frequency range between microwaves and visible light. These waves are beneficial for addressing dermatologic issues. The extent to which radiation penetrates the skin depends on factors such as vascularity, radiation wavelength, skin structure, and pigmentation. Infrared radiation is employed in heat therapy, where it directly warms the blood capillaries in the affected area of the human body. This process increases blood circulation, aiding in the treatment of superficial wound infections, boosting white blood cell count, and eliminating waste products^25–39^:

The above cited published works certainly indicate the significance of present mathematical model and it is evident that the cilia propelled flow considering an integrated analysis on symmetric plus asymmetric conduit is not mathematically modeled yet. We have incorporated the Couple stress non-Newtonian model with its physiological applications under the interaction of elecro osmosis, heat transfer and thermal radiation. A descriptive graphical illustration is conveyed for both of these models and physical outcomes are presented. Since we have modeled this problem for symmetric plus asymmetric conduit, therefore a cartesian coordinates system is established for current analysis to get exact solutions in the end. The composite impact of peristaltic flow in addition to cilia driven metachronal wave effect is depicted. Streamlines convey the flow visualization for both symmetric beside asymmetric conduit.

## Mathematical formulation

**Figure 1:**
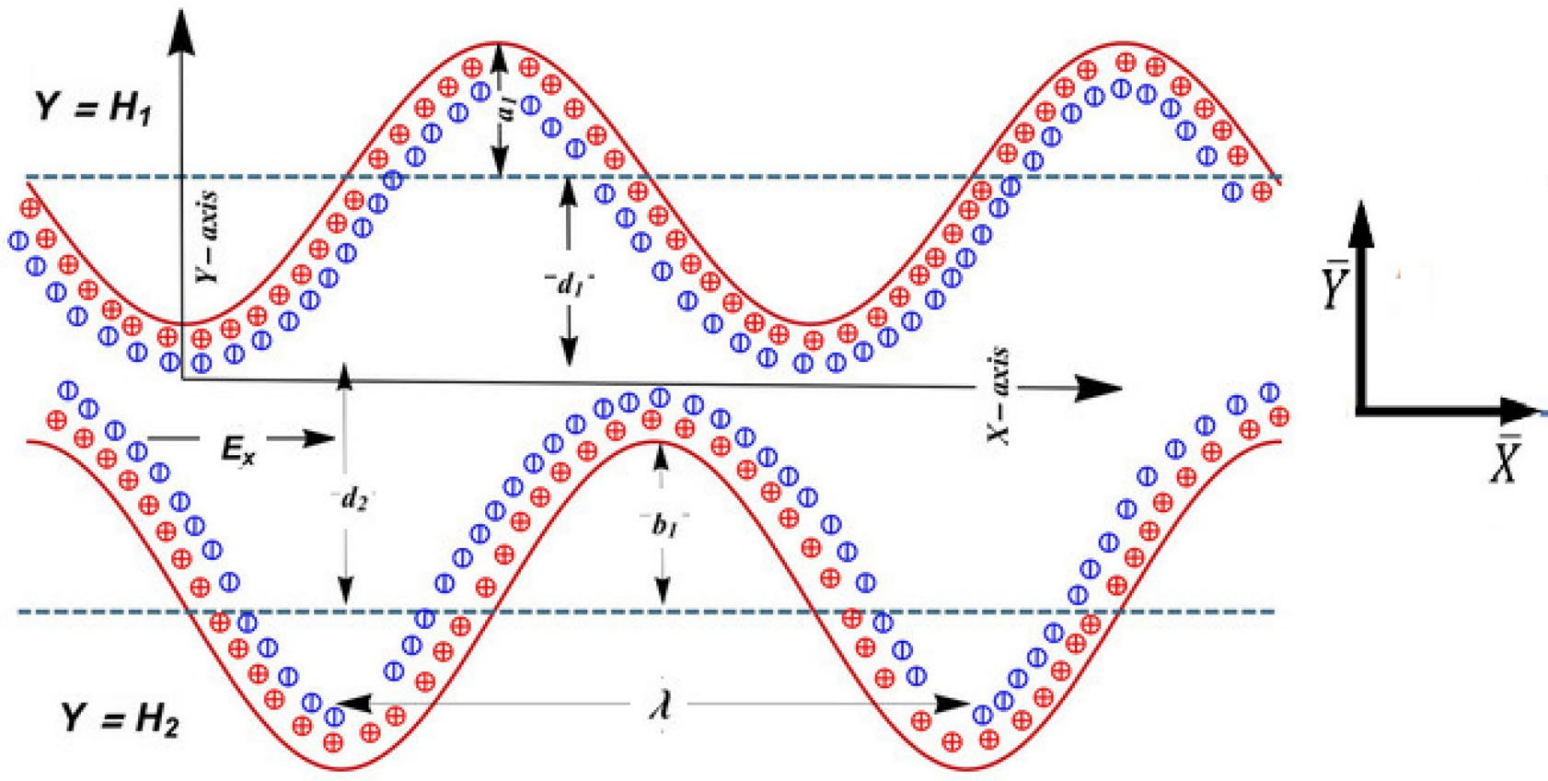
Geometry of the problem.

We have incorporated the cartesian coordinate system to mathematically establish a model that provides the integrated analysis on symmetric plus asymmetric conduit flow of non-Newtonian fluids. This research discloses a combined analysis on metachronal cilia propulsion plus peristaltic flow mechanism for a couple stress non-Newtonian model. The geometrical configuration for such a flow model is given as in see Fig. 1.

The activity of cilia in elliptical tracks is mathematically configurated as^18,19^

1 $${{\bar{Y}}}=f\left({{\bar{X}}},t\right)=\pm \left[a+a\varepsilon {\cos}\left(\frac{2\pi }{\lambda }\left({{\bar{X}}}-{c}_{1}t\right)\right)\right]=\pm L=\pm H,$$

2 $${{\bar{X}}}=g\left({{\bar{X}}},t\right)={X}_{0}+a\varepsilon \alpha {\sin}\left(\frac{2\pi }{\lambda }\left({{\bar{X}}}-{c}_{1}t\right)\right),$$

Further, this cilia activity takes some horizontal plus vertical velocities given below

3 $${U}_{0}=\frac{-\left(\frac{2\pi }{\lambda }\right)a\varepsilon \alpha {c}_{1}{\cos}\left(\frac{2\pi }{\lambda }\left({{\bar{X}}}-{c}_{1}t\right)\right)}{1-\left(\frac{2\pi }{\lambda }\right)a\varepsilon \alpha {c}_{1}{\cos}\left(\frac{2\pi }{\lambda }\left({{\bar{X}}}-{c}_{1}t\right)\right)},$$

4 $${V}_{0}=\frac{-\left(\frac{2\pi }{\lambda }\right)a\varepsilon \alpha {c}_{1}{\sin}\left(\frac{2\pi }{\lambda }\left({{\bar{X}}}-{c}_{1}t\right)\right)}{1-\left(\frac{2\pi }{\lambda }\right)a\varepsilon \alpha {c}_{1}{\sin}\left(\frac{2\pi }{\lambda }\left({{\bar{X}}}-{c}_{1}t\right)\right)}.$$

The problem being analyzed is a steady flow problem under the frame conversions given as

5 $${{\bar{X}}}={{\bar{X}}}-ct,\hspace{0.33em}\overline{y}={{\bar{Y}}},\hspace{0.33em}{{\bar{u}}}={{\bar{u}}}-c,\hspace{0.33em}{{\bar{v}}}={{\bar{v}}},\hspace{0.33em}{{\bar{P}}}\left({{\bar{X}}}\right)=\overline{P}\left({{\bar{X}}},t\right).$$

The non-Newtonian nature of couple stress fluid is linked with the present problem by utilizing the following equations

6 $${T}_{ji}{,}_{j}=\rho \frac{\partial {v}_{i}}{\partial t},$$

7 $${e}_{ijk}{T}_{jk}^{A}+{M}_{ji}{,}_{j}=0,$$

8 $${T}_{ij}=-{{\bar{P}}}{\delta }_{ij}+2\mu {d}_{ij},$$

9 $${\mu }_{ij}=4\eta {w}_{j,i}+\eta {w}_{i,j},$$

After utilizing Eqs. (6–9) in our governing flow equations, we are left with the following simplified equations

10 $$\frac{\partial {{\bar{u}}}}{\partial {{\bar{X}}}}+\frac{\partial {{\bar{v}}}}{\partial {{\bar{Y}}}}=0$$

11 $$\rho \left(\frac{\partial {{\bar{u}}}}{\partial {{\bar{X}}}}+{{\bar{v}}}\frac{\partial {{\bar{u}}}}{\partial {{\bar{Y}}}}\right)=-\frac{\partial {{\bar{P}}}}{\partial {{\bar{X}}}}+\mu \hspace{0.33em}{\nabla }^{2}{{\bar{u}}}-\eta \hspace{0.33em}{\nabla }^{4}{{\bar{u}}}+{\rho }_{e}{E}_{{{\bar{X}}}},$$

12 $$\rho \left({{\bar{u}}}\frac{\partial {{\bar{v}}}}{\partial {{\bar{X}}}}+{{\bar{v}}}\frac{\partial {{\bar{v}}}}{\partial {{\bar{Y}}}}\right)=-\frac{\partial {{\bar{P}}}}{\partial {{\bar{Y}}}}+\mu \hspace{0.33em}{\nabla }^{2}{{\bar{v}}}-\eta \hspace{0.33em}{\nabla }^{4}{{\bar{v}}},$$

13 $$ \begin{aligned}  \rho \left({{\bar{u}}}\frac{\partial {{\bar{T}}}}{\partial {{\bar{X}}}}+{{\bar{v}}}\frac{\partial {{\bar{T}}}}{\partial {{\bar{Y}}}}\right) & =k\hspace{0.33em}{\nabla }^{2}{{\bar{T}}}+\tau .{L}^{t}-\frac{\partial {q}_{r}}{\partial {{\bar{Y}}}}, \\  {\nabla }^{2} &=\frac{{\partial }^{2}}{\partial {{{\bar{X}}}}^{2}}+\frac{{\partial }^{2}}{\partial {{{\bar{Y}}}}^{2}}, \, \hspace{0.33em}{\nabla }^{4}={\nabla }^{2}\hspace{0.33em}{\nabla }^{2} \end{aligned} $$

$\tau .{L}^{t}$ is a viscous dissipation term define below

$$\upsilon \left({\left(\frac{\partial {{\bar{u}}}}{\partial {{\bar{X}}}}\right)}^{2}+{{\bar{2}}}{\left(\frac{\partial {{\bar{v}}}}{\partial {{\bar{Y}}}}\right)}^{2}+{\left(\frac{\partial {{\bar{u}}}}{\partial {{\bar{X}}}}+\frac{\partial {{\bar{v}}}}{\partial {{\bar{Y}}}}\right)}^{2}\right)+\frac{\eta \hspace{0.33em}}{\rho } \left({\left(\frac{{\partial }^{2}{{\bar{u}}}}{\partial {{{\bar{X}}}}^{2}}+\frac{{\partial }^{2}{{\bar{u}}}}{\partial {{{\bar{Y}}}}^{2}}\right)}^{2}+{\left(\frac{{\partial }^{2}{{\bar{v}}}}{\partial {{{\bar{X}}}}^{2}}+\frac{{\partial }^{2}{{\bar{v}}}}{\partial {{{\bar{Y}}}}^{2}}\right)}^{2}\right),$$

The non-dimensional formation of present problem is availed by engaging the subsequent dimensionless parameters

14 $$\begin{aligned} y &=\frac{{{\bar{Y}}}}{a}, x=\frac{{{\bar{X}}}}{\lambda }, t=\frac{c{{\bar{T}}}}{\lambda }, v=\frac{\lambda {{\bar{v}}}}{ac}, \varepsilon =\frac{b}{a}, u=\frac{{{\bar{u}}}}{c}, \\ \theta & =\frac{{{\bar{T}}}-{{{\bar{T}}}}_{0}}{{{{\bar{T}}}}_{0}},\theta =\frac{{{\bar{T}}}-{{{\bar{T}}}}_{0}}{{{{\bar{T}}}}_{1}-{{{\bar{T}}}}_{0}}, P=\frac{{{\bar{P}}}{a}^{2}}{\lambda c\mu }, h=\frac{{{\bar{h}}}}{a}, {\Re}=\frac{\rho ca}{\mu }, \beta =\frac{a}{\lambda } . \end{aligned}$$

By applying the lubrication approximation theory, we can express the flow equations in a dimensionless form yield.

15 $$\frac{\partial P}{\partial x}=\frac{{\partial }^{2}u}{\partial {y}^{2}}-\frac{1}{{\xi }^{2}}\frac{{\partial }^{4}u}{\partial {y}^{4}}+{U}_{HS}\frac{{\partial }^{2}E}{\partial {y}^{2}}$$

16 $$\frac{\partial P}{\partial y}=0,$$

17 $$\frac{{\partial }^{2}\theta }{\partial {y}^{2}}+Pr{E}_{c}\left({\left(\frac{\partial u}{\partial y}\right)}^{2}+\frac{1}{{\xi }^{2}}{\left(\frac{{\partial }^{2}u}{\partial {y}^{2}}\right)}^{2}\right)+PrR\frac{{\partial }^{2}\theta }{\partial {y}^{2}},$$

18 $$\frac{{\partial }^{2}E}{\partial {y}^{2}}={\upkappa }^{2}\left(\frac{{\mathrm{n}}^{-}-{\mathrm{n}}^{+}}{2}\right),$$

where ${U}_{HS}$ designates the Helmholtz-Smoluchowski velocity or electroosmotic velocity parameter, $Pr$ the Prandtl number, $R$ the dimensionless thermal radiation parameter, $\theta$ the dimensionless temperature parameter, and $\kappa$ is the ratio of the characteristic traverse length to the Debye length parameter. The local ionic distribution of ionic species can be specified by linearized Boltzmann distribution for low zeta potential which accurately estimates the electric potential established in the fluid medium without increasing the complexity of the flow problem as for most of the electrolyte solution, the generated electric potential lies in the range less than or equal to 25 mV.

19 $${n}^{\pm }={e}^{\mp E},$$

After the linearized Poisson-Boltzmann paradigm^31^ as:

20 $$\frac{{\partial }^{2}E}{\partial {y}^{2}}={\kappa }^{2}\mathrm{sinh}\left(E\right),$$

which is further simplified under Debye-Hückel approximation^31^ i.e. $\mathrm{sinh}\left(\varphi \right)\approx \varphi$ as:

21 $$\frac{{\partial }^{2}E}{\partial {y}^{2}}={\kappa }^{2}E.$$

The dimensionless form of the no-slip boundary conditions for velocity temperature and the symmetric conduit case incorporates the subsequent conditions

22 $$\frac{\partial u}{\partial y}=0, \frac{{\partial }^{3}u}{\partial {y}^{3}}=0, \, \frac{\partial \theta }{\partial y}=0, \frac{\partial E}{\partial y}=0, \text{at }y=0,$$

23 $$u=-1-\frac{2\pi \varepsilon \alpha \beta \mathit{cos}\left(x\right)}{1-2\pi \varepsilon \alpha \beta \mathit{cos}\left(x\right)}, \frac{{\partial }^{2}u}{\partial {y}^{2}}=0,E=\xi , \theta =0\hspace{0.33em}\text{ at }y=h=1+\varepsilon \mathit{cos}\left(x\right),$$

24 $$u=-1-\frac{2\pi \varepsilon \alpha \beta \mathit{cos}\left(x\right)}{1-2\pi \varepsilon \alpha \beta \mathit{cos}\left(x\right)}, \frac{{\partial }^{2}u}{\partial {y}^{2}}=0, \theta =0,E=\xi , \text{ at }y={h}_{1}=1+\varepsilon \mathit{cos}\left(x\right)$$

25 $$u=-1-\frac{2\pi \varepsilon \alpha \beta \mathit{cos}\left(x\right)}{1-2\pi \varepsilon \alpha \beta \mathit{cos}\left(x\right)}, \frac{{\partial }^{2}u}{\partial {y}^{2}}=0,\theta =1, E=\xi , \hspace{0.33em}\text{ at }y={h}_{2}=-d-b\mathit{cos}\left(x+\varphi \right),$$

The asymmetric conduit case incorporates the subsequent conditions.

## Computational procedure

**Figure 2:**
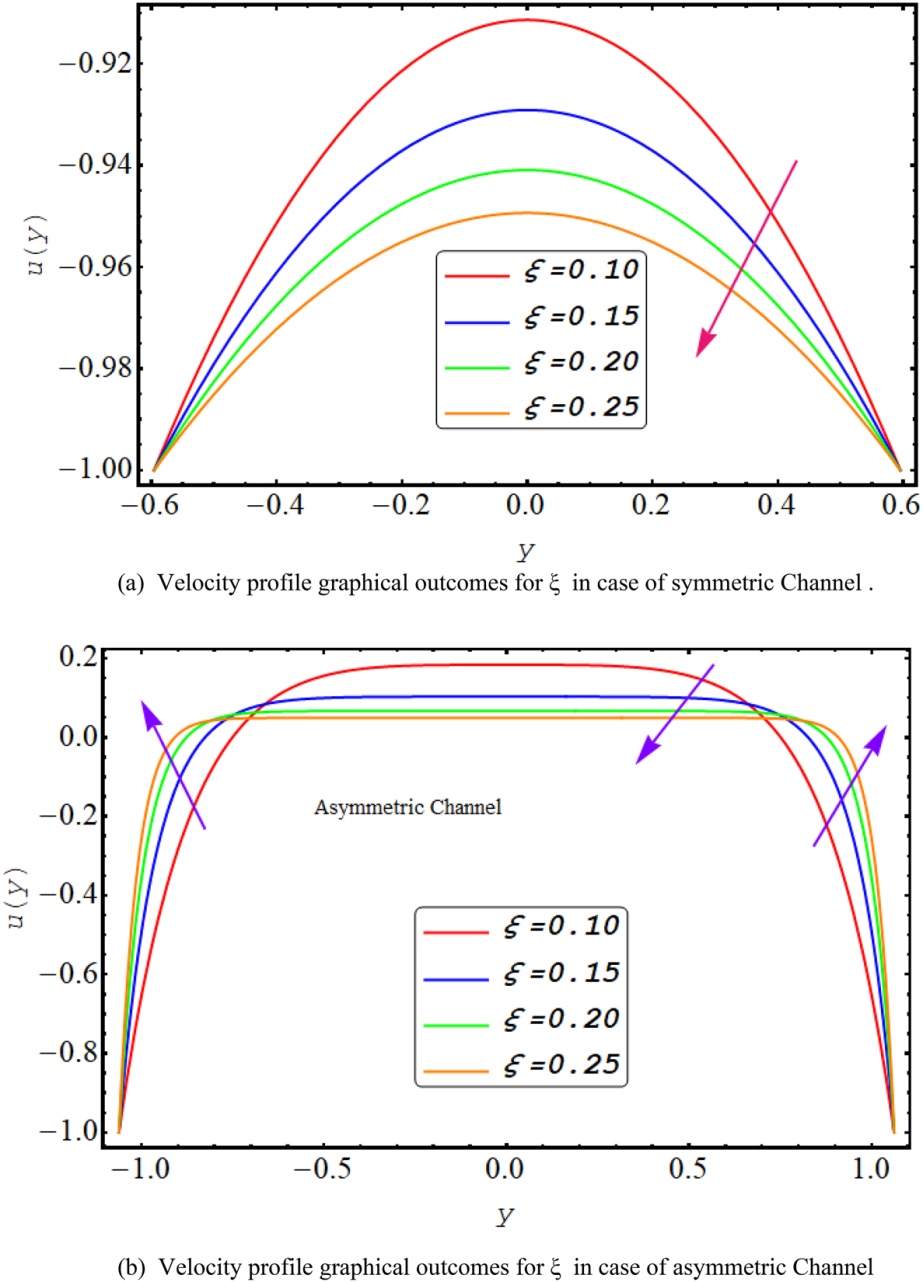
(**a**) Velocity profile graphical outcomes for ξ in case of symmetric channel. (**b**) Velocity profile graphical outcomes for ξ in case of asymmetric channel.


To solve the simplified system defined in Eqs. (15) to (21) owing to boundary conditions in Eqs. (22) to (25). Computational software MATLAB is utilized, and numerical results are obtained by employing inbuilt solver based of three stage Lobatto IIIa formula known as bvp4c. Figure 2 illustrate the complete flow chart of bvp4c algorithm.

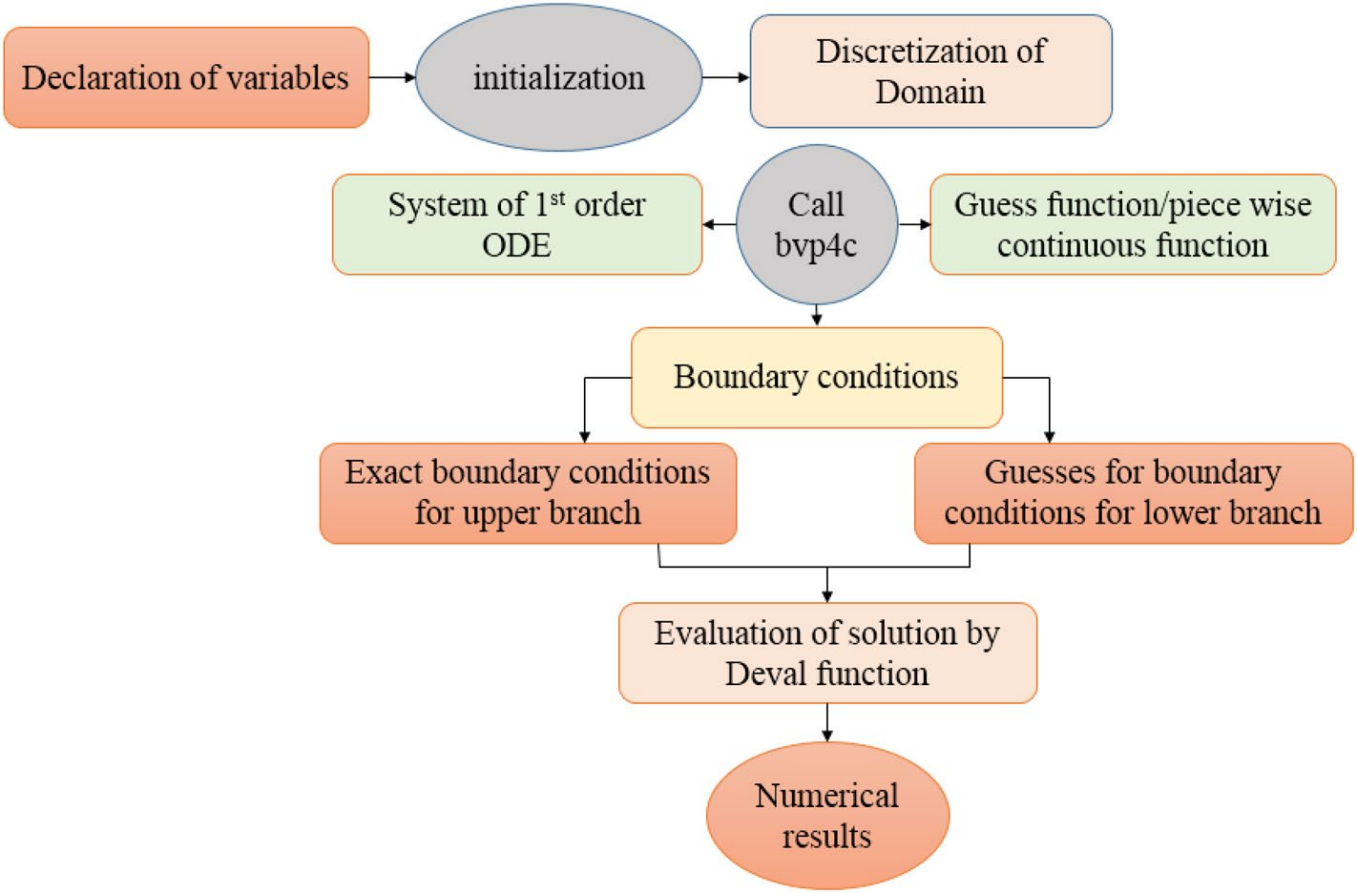


## Results and discussion

**Figure 3:**
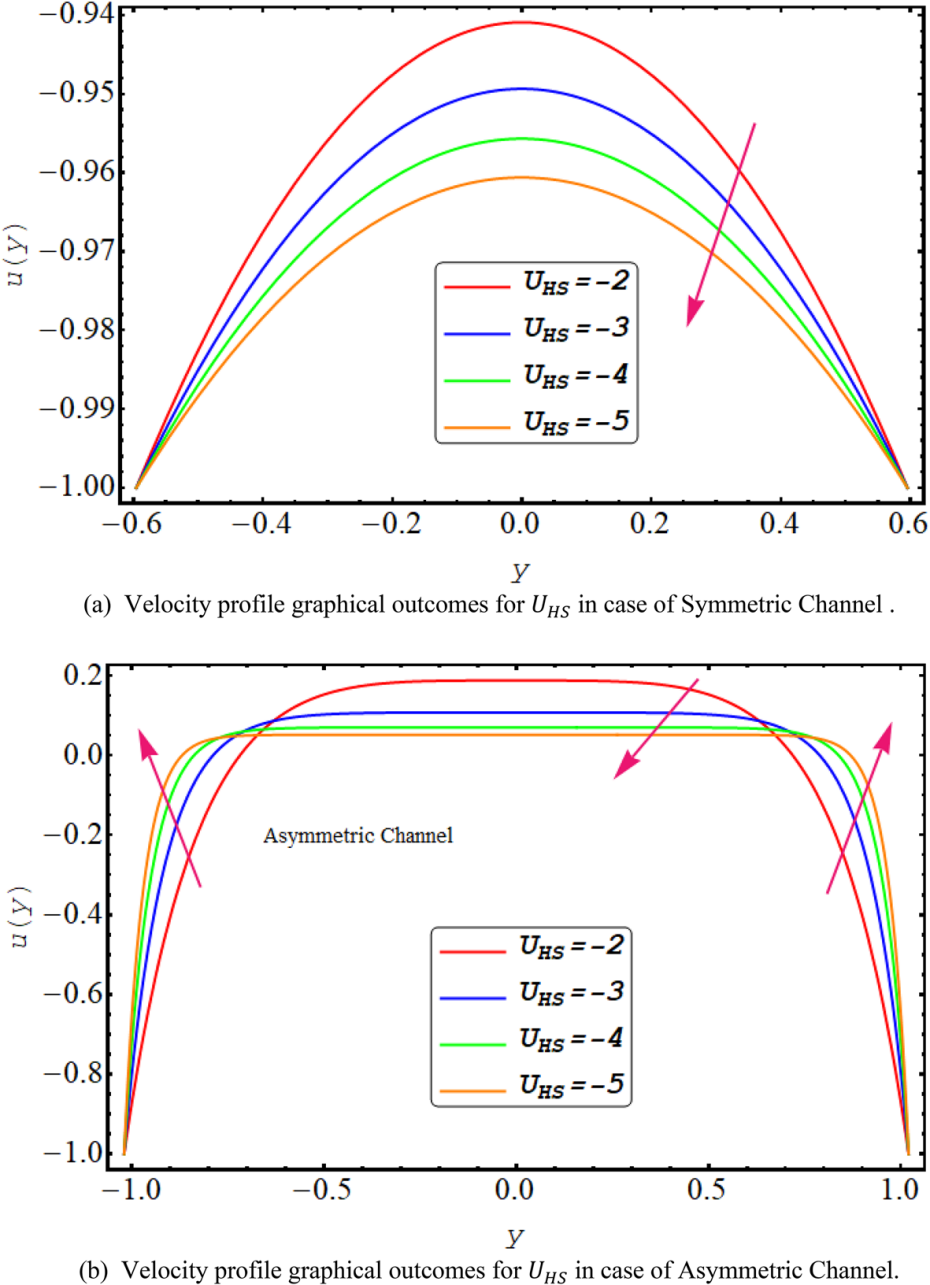
(**a**). Velocity profile graphical outcomes for ${U}_{HS}$ in case of symmetric channel. (**b**) Velocity profile graphical outcomes for ${U}_{HS}$ in case of asymmetric channel.

**Figure 4:**
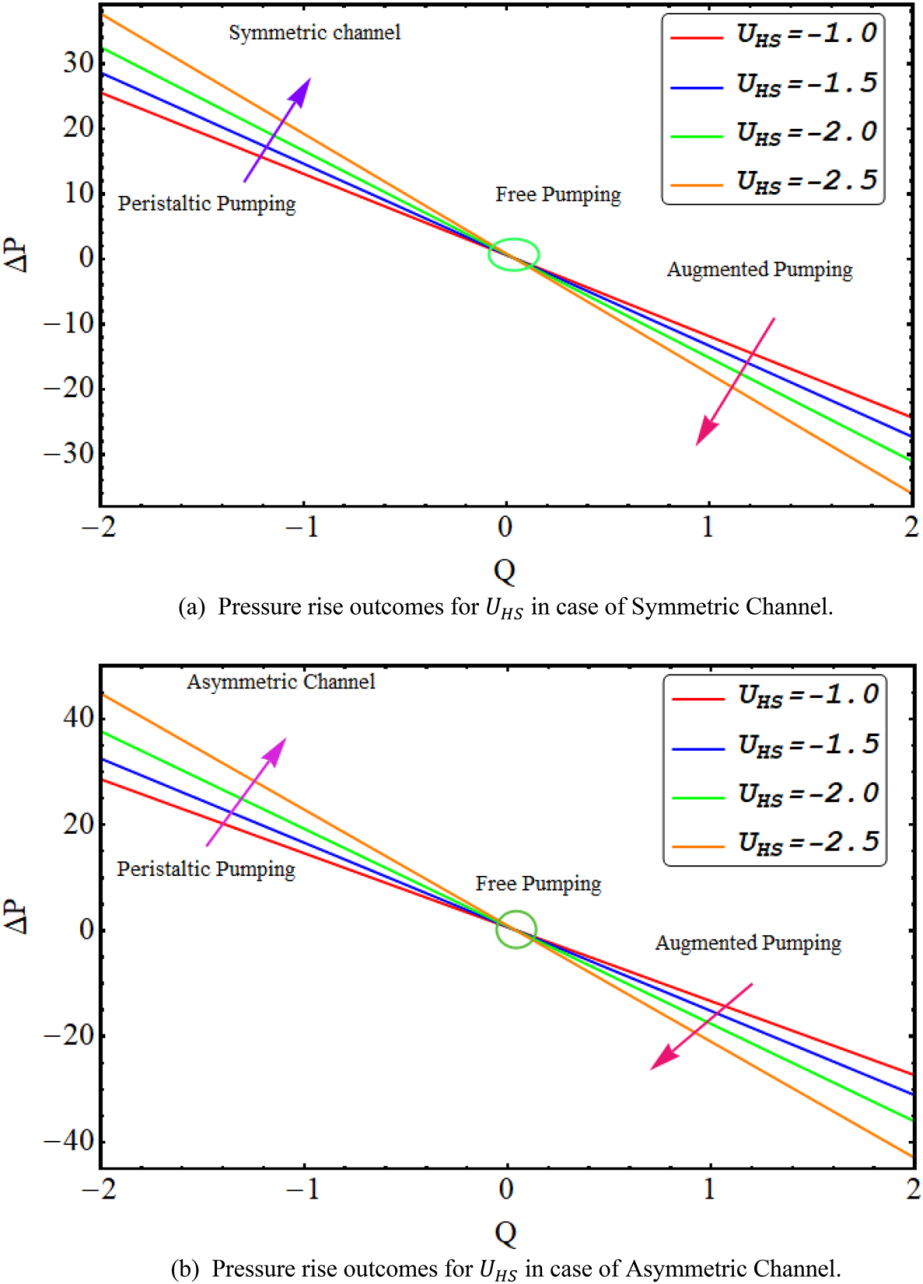
(**a**) Pressure rise outcomes for ${U}_{HS}$ in case of symmetric channel. (**b**) Pressure rise outcomes for ${U}_{HS}$ in case of asymmetric channel.

**Figure 5:**
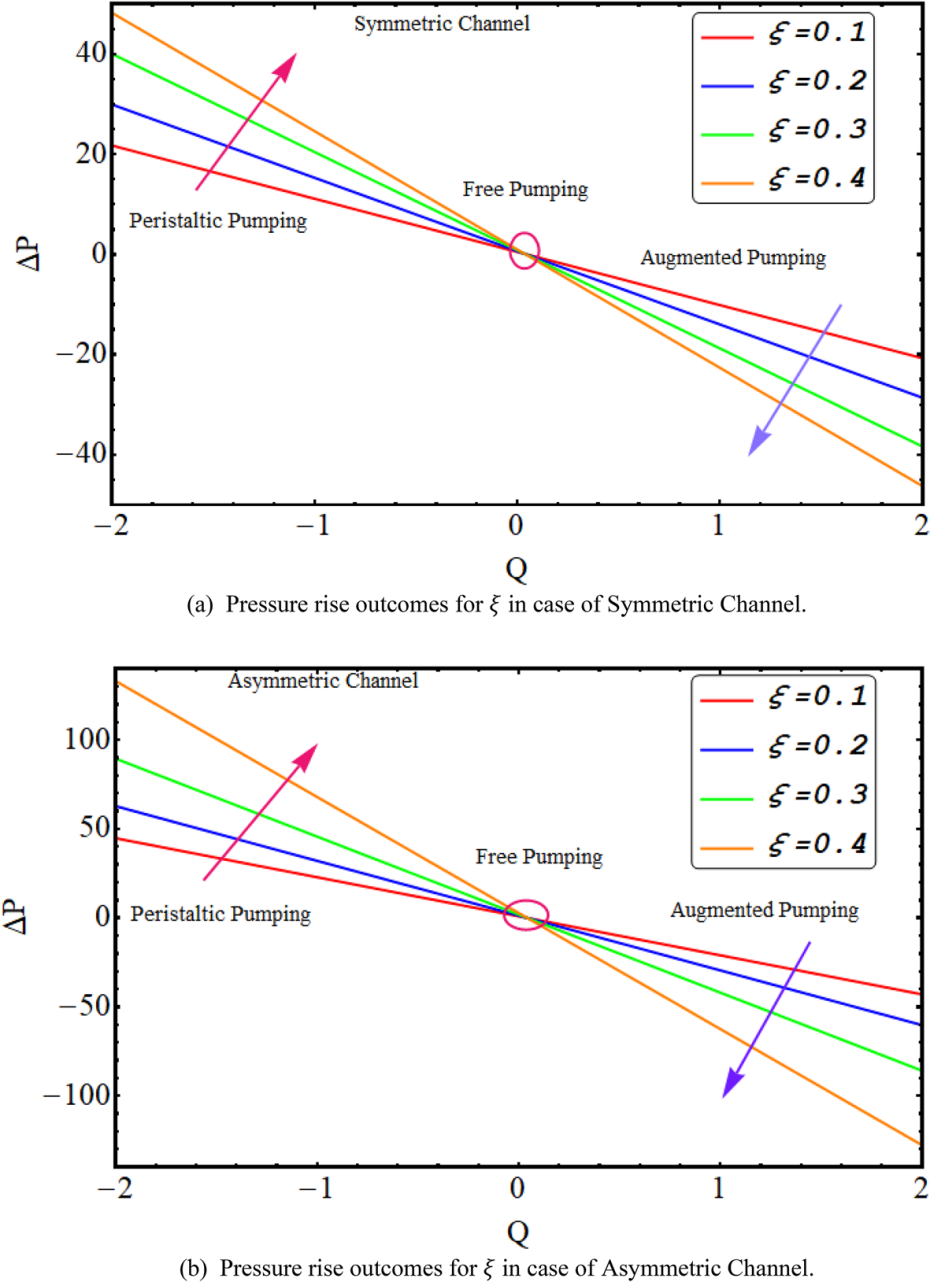
(**a**) Pressure rise outcomes for $\xi$ in case of symmetric channel. (**b**) Pressure rise outcomes for $\xi$ in case of asymmetric channel.

In this division, the graphical demonstrations of above mathematical calculations are presented. An integrated graphical analysis is established for both the considered cases of symmetric in addition to asymmetric conduit. The flow profile (velocity), pressure gradient for both cases, numerically computed $\Delta P$ results for both cases, Temperature profile and finally streamlines are disclosed graphically. Figure 2 exhibit the velocity flow profile for dimensionless quantity of couple stress fluid parameter $\xi$ symmetric and asymmetric channel respectively. Figure 2a shows that velocity is declining for symmetric conduit case with the rise in couple stress fluid parameter $\xi$ due to high shear rate viscosity of couple stress fluid in addition to asymmetric conduit case with an incrementing value of $\xi$ velocity profile decreases at the center of the channel but near the wall of the channel velocity profile increases due to low wave amplitude see Fig. 2b. Figure 3 reveals that velocity, for both cases of symmetric as well as asymmetric conduit, is a decreasing function of Helmholtz–Smoluchowski velocity ${U}_{HS}$ as increase in ${U}_{HS}$ negative direction physically implies the presence of a strong electric field which produces an enhancement in the velocity profile although for asymmetric case near the walls of the channel velocity profile increases due to change in amplitude. Further it is revealed that the asymmetric conduit attains a higher flow profile in relation to the symmetric conduit. Figure 4 disclose the numerical computations performed for $\Delta P$ against $Q$ for Helmholtz–Smoluchowski velocity ${U}_{HS}$ for both symmetric and asymmetric conduit case respectively. Here $\Delta P$ is a rising function of ${U}_{HS}$ for both conduit cases, revealed in Fig. 4 for peristaltic pumping region and in augmented pumping region opposite behavior is obvious as increase in ${U}_{HS}$ negative direction physically implies the presence of a strong electric field which produces an enhancement in the velocity profile that rises the pressure rise. A rapid enhance in numerical values of $\Delta P$ is noted for asymmetric conduit case in relation to the symmetric conduit. Further, $\Delta P$ is an incrementing function of ξ, shown by Fig. 5 in peristaltic pumping region ad decreases in augmented pumping region due to high shear rate viscosity of couple stress fluid. Again, it is seen that $\Delta P$ has high numerical values of asymmetric conduit. Moreover, $\Delta P=0$ region is observed in these numerical computations that refers to the free pumping zone.

**Figure 6:**
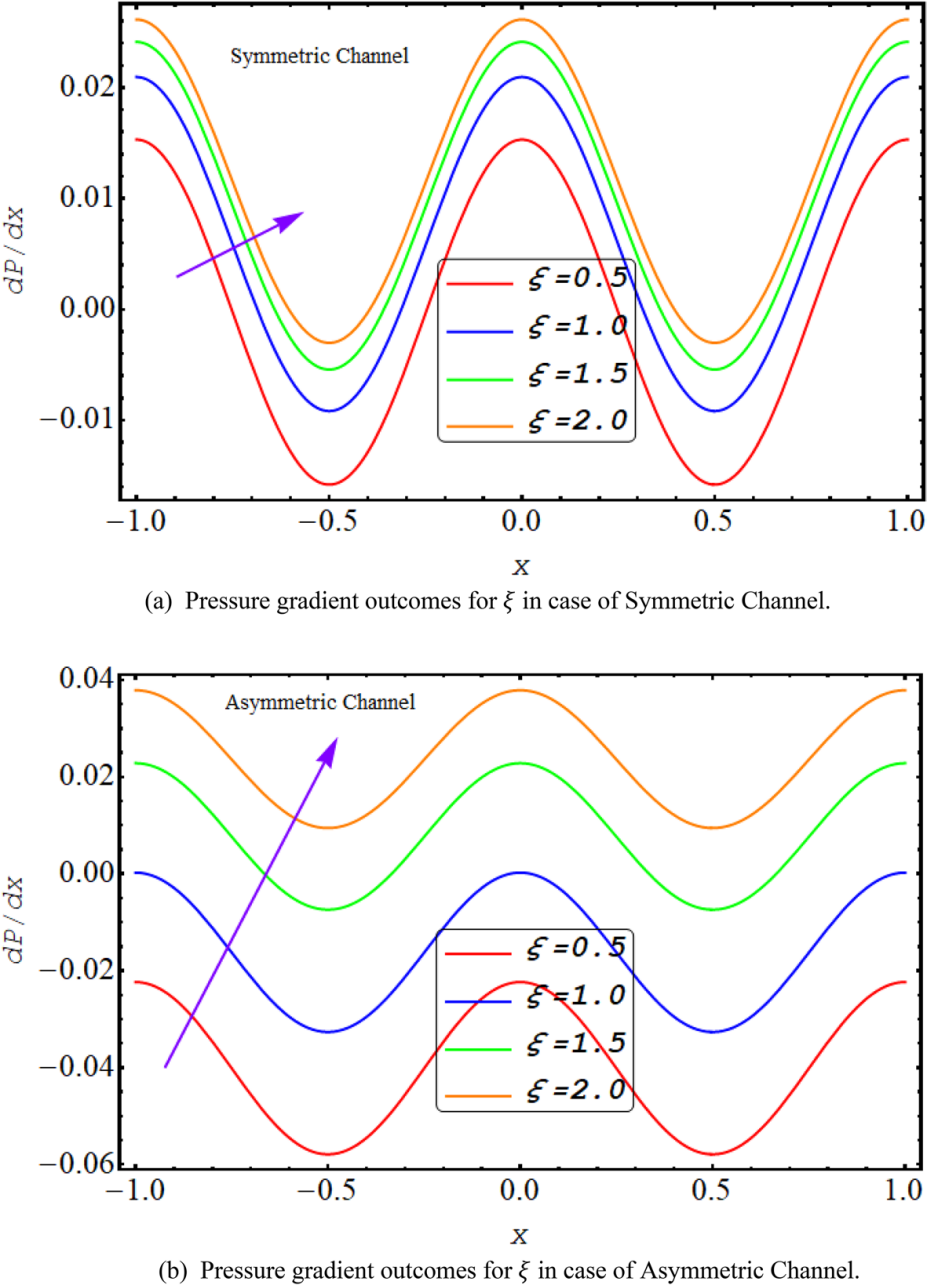
(**a**) Pressure gradient outcomes for $\xi$ in case of symmetric channel. (**b**). Pressure gradient outcomes for $\xi$ in case of asymmetric channel.

**Figure 7:**
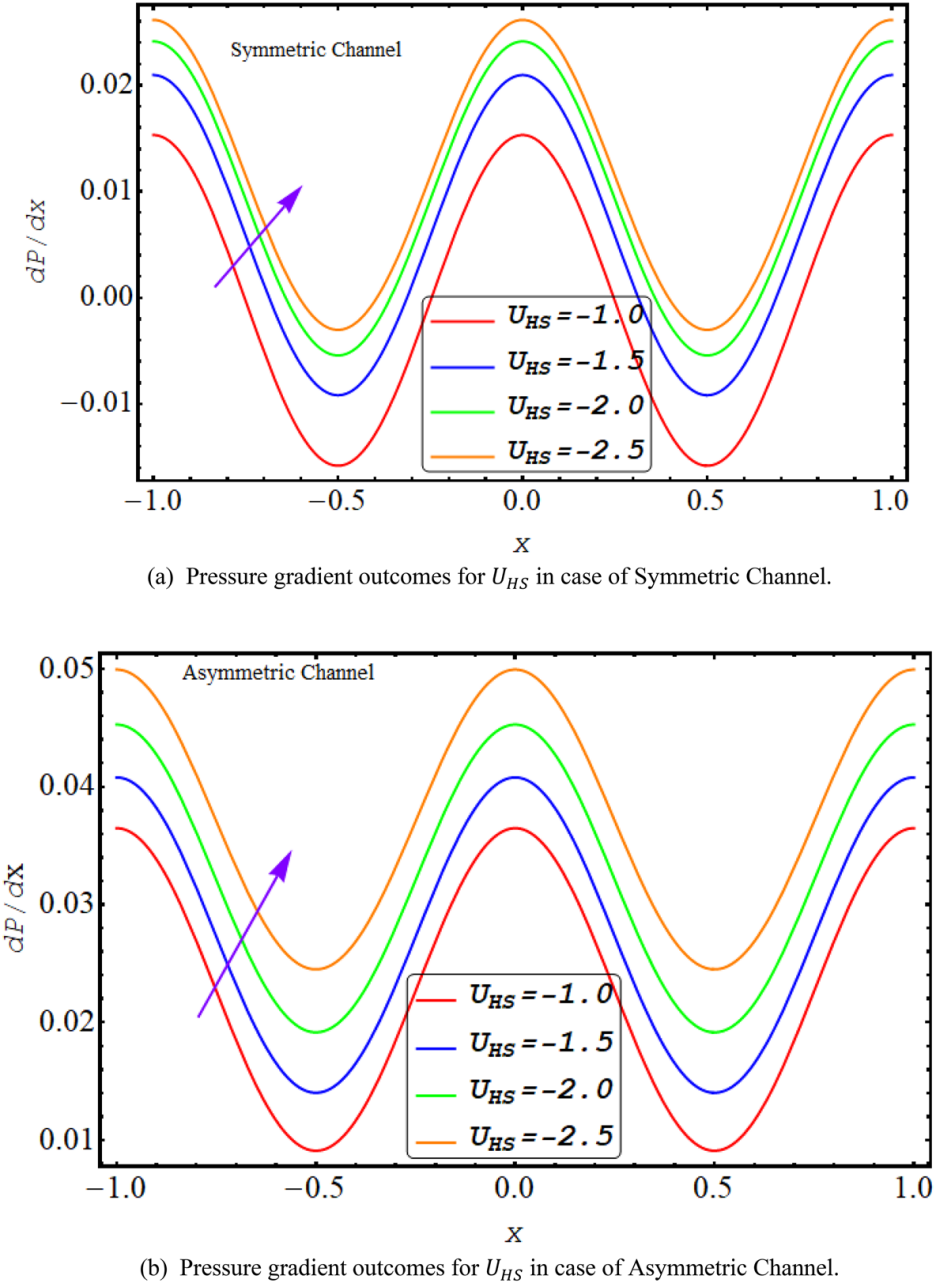
(**a**) Pressure gradient outcomes for ${U}_{HS}$ in case of symmetric channel. (**b**) Pressure gradient outcomes for ${U}_{HS}$ in case of asymmetric channel.

Figures 6 and 7 exhibit the graphical results of $\frac{dp}{dx}$ plotted for $\xi$ and ${U}_{HS}$ for both symmetric and asymmetric conduit respectively. Its reveals from figures that for rise in $\xi$ and ${U}_{HS}$ value of $\frac{dp}{dx}$ for both cases of asymmetric or symmetric conduit increases. As increase in ${U}_{HS}$ negative direction physically implies the presence of a strong electric field which produces an enhancement in the velocity profile that rises the pressure gradient. Further high shear rate of couple stress fluid also increases pressure gradient. Maximum pressure gradient is in the central section of conduit while they decline towards walls.

**Figure 8:**
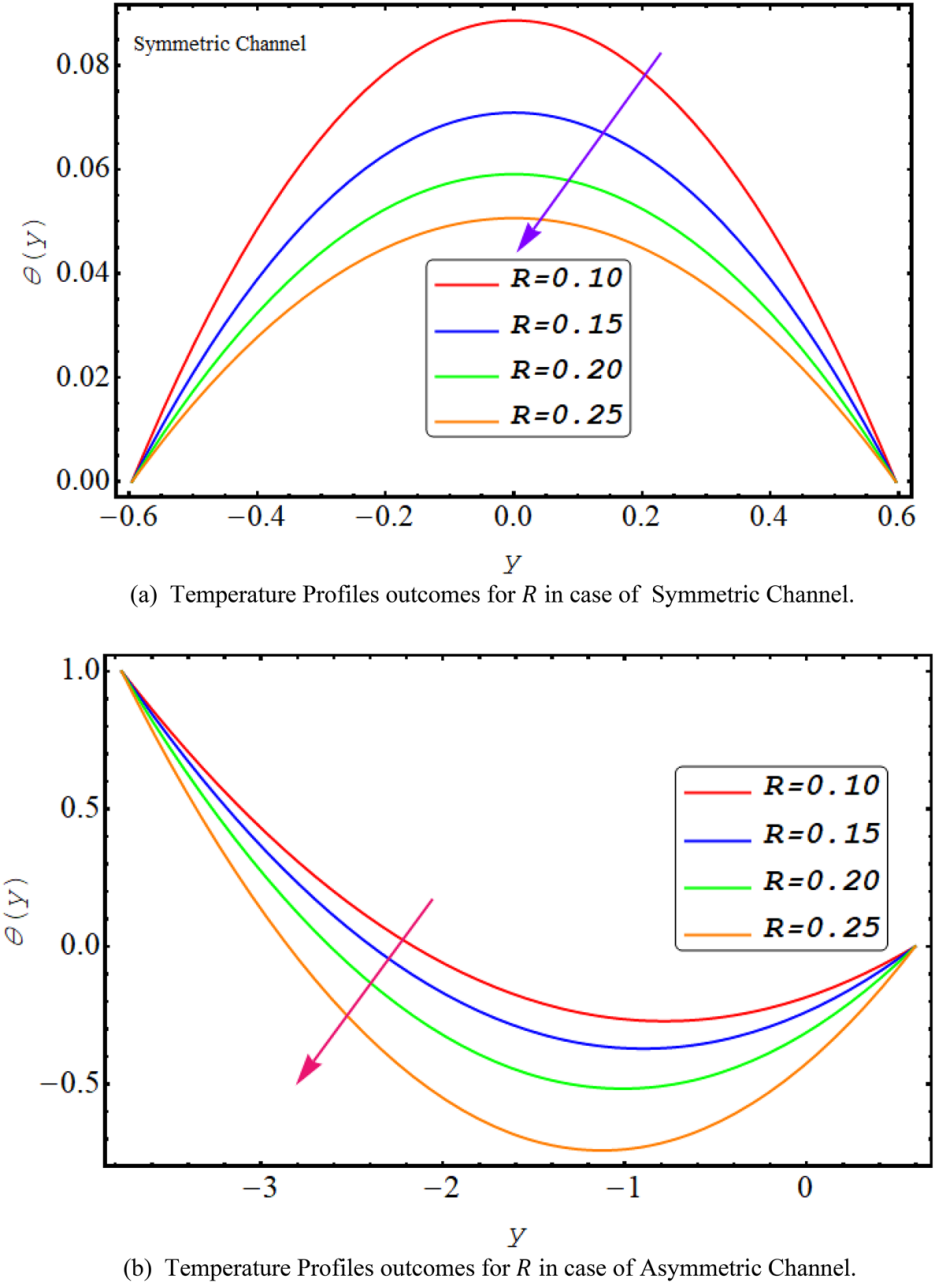
(**a**). Temperature profiles outcomes for $R$ in case of symmetric channel. (**b**) Temperature profiles outcomes for $R$ in case of asymmetric channel.

**Figure 9:**
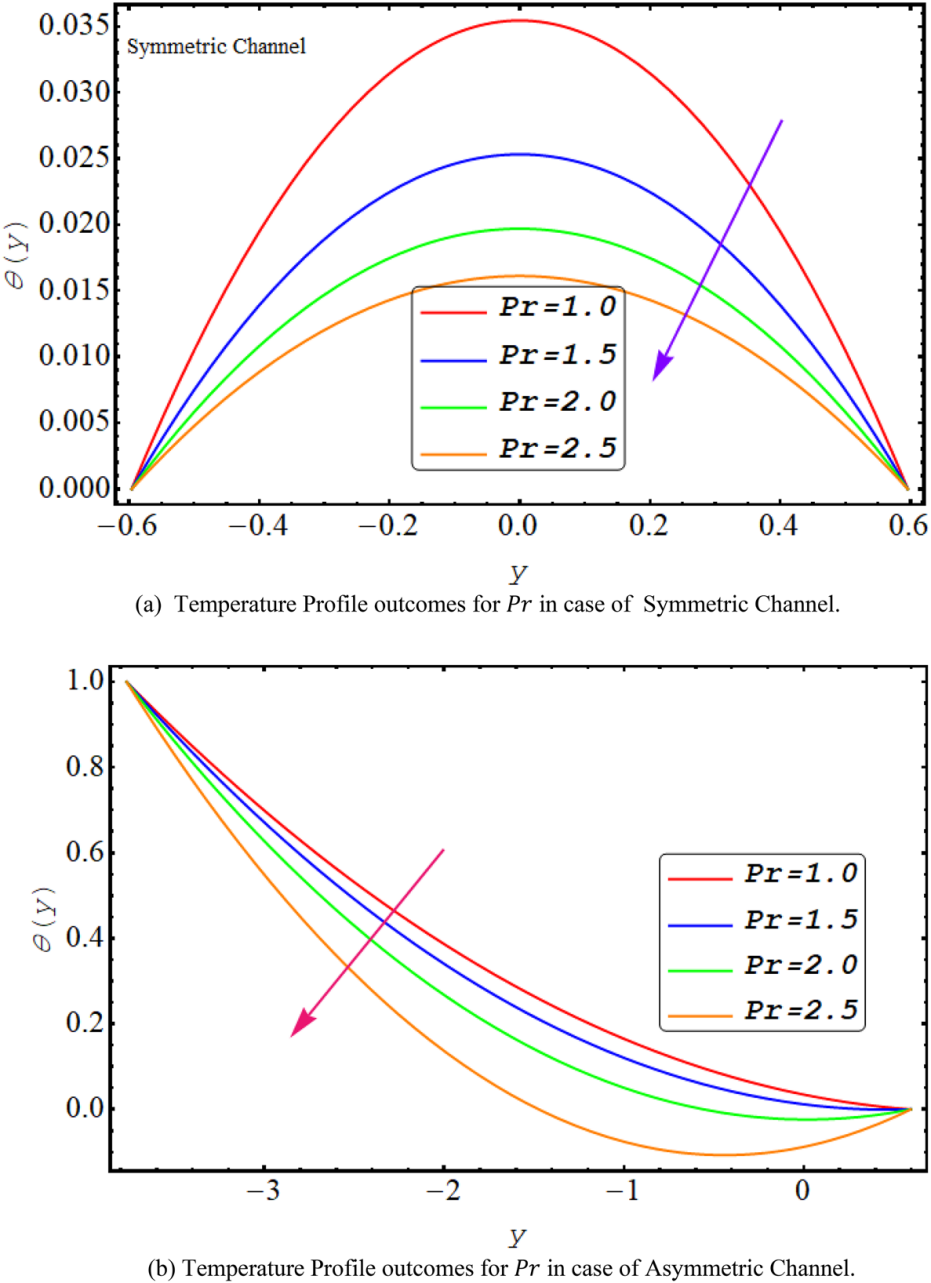
(**a**) Temperature profile outcomes for $Pr$ in case of symmetric channel. (**b**) Temperature profile outcomes for $Pr$ in case of asymmetric channel.

**Figure 10:**
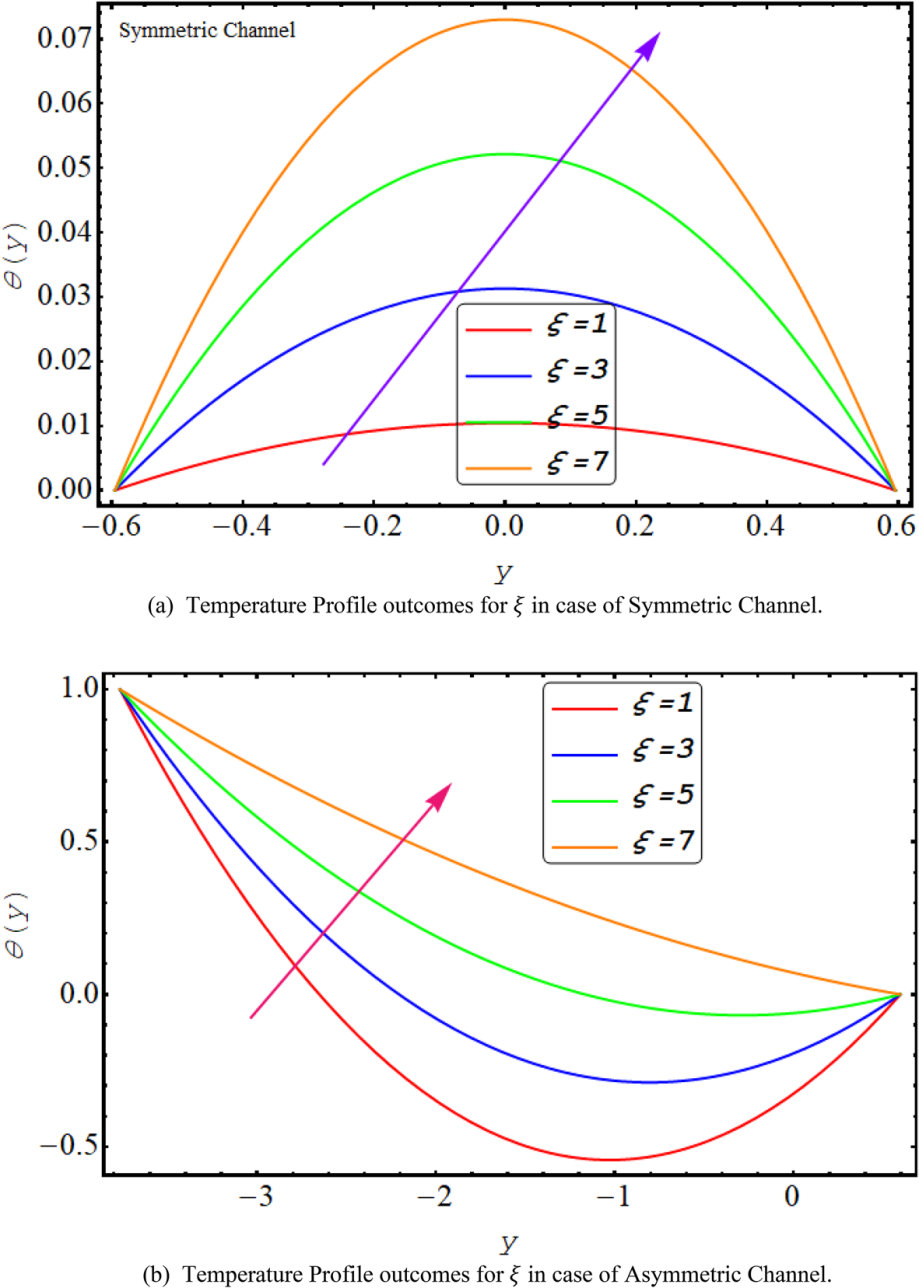
(**a**) Temperature profile outcomes for $\xi$ in case of symmetric channel. (**b**) Temperature Profile outcomes for $\xi$ in case of asymmetric channel.

Figure 8 shows a decreasing temperature profile for both asymmetric or symmetric conduit with an increase in thermal radiation. This is due to electromagnetic radiation generated by the thermal motion of particles in matter. It is observed that the temperature profile decreases with the rise in Prandtl number because it is viscosity of a fluid in correlation with the thermal conductivity and high viscosity reduces temperature profile see Fig. 9. Figure 10 presents that due to high shear rate of couple stress fluid temperature profile increases with the increase in couple stress fluid parameter for both symmetric and asymmetric case.

**Figure 11:**
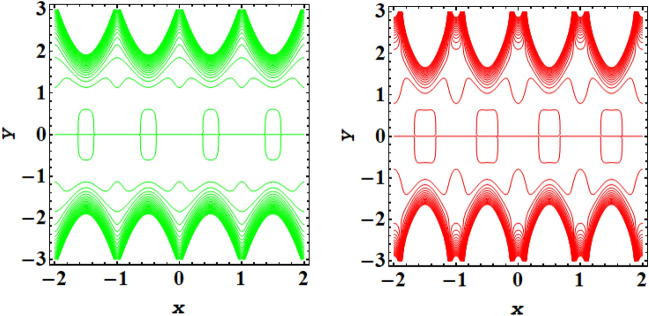
Streamlines outcomes for symmetric channel when $\xi$=0.4, 0.8.

**Figure 12:**
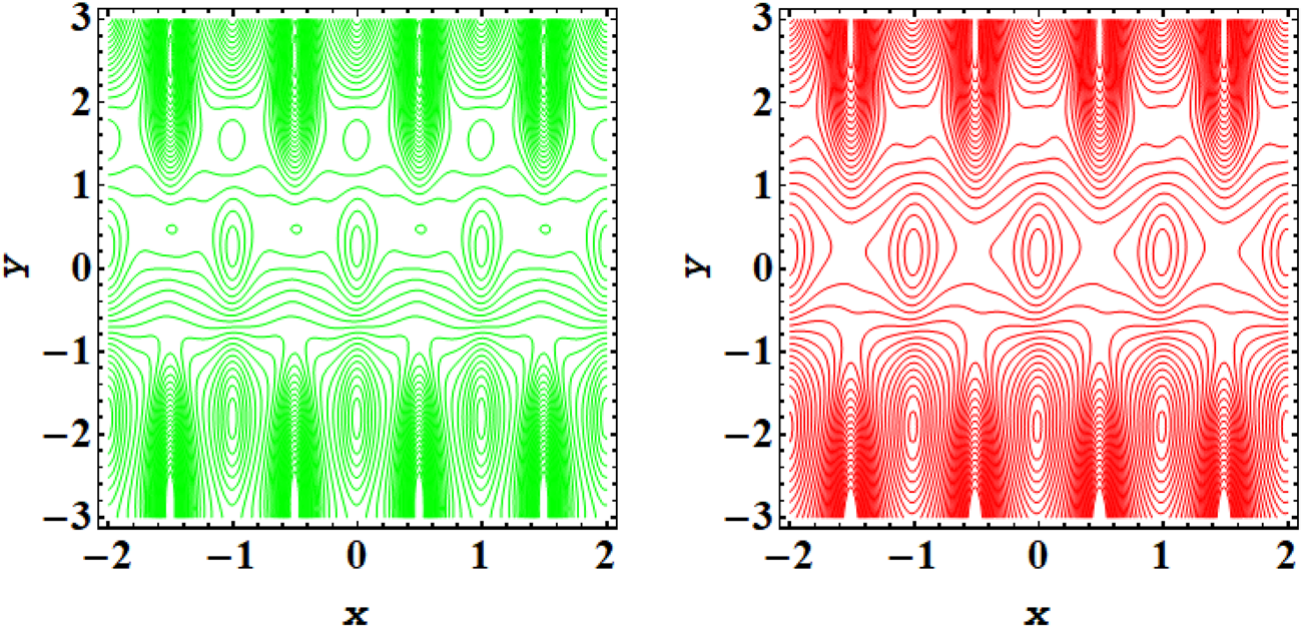
Streamlines outcomes for asymmetric channel when $\xi$=0.4, 0.8.

**Figure 13:**
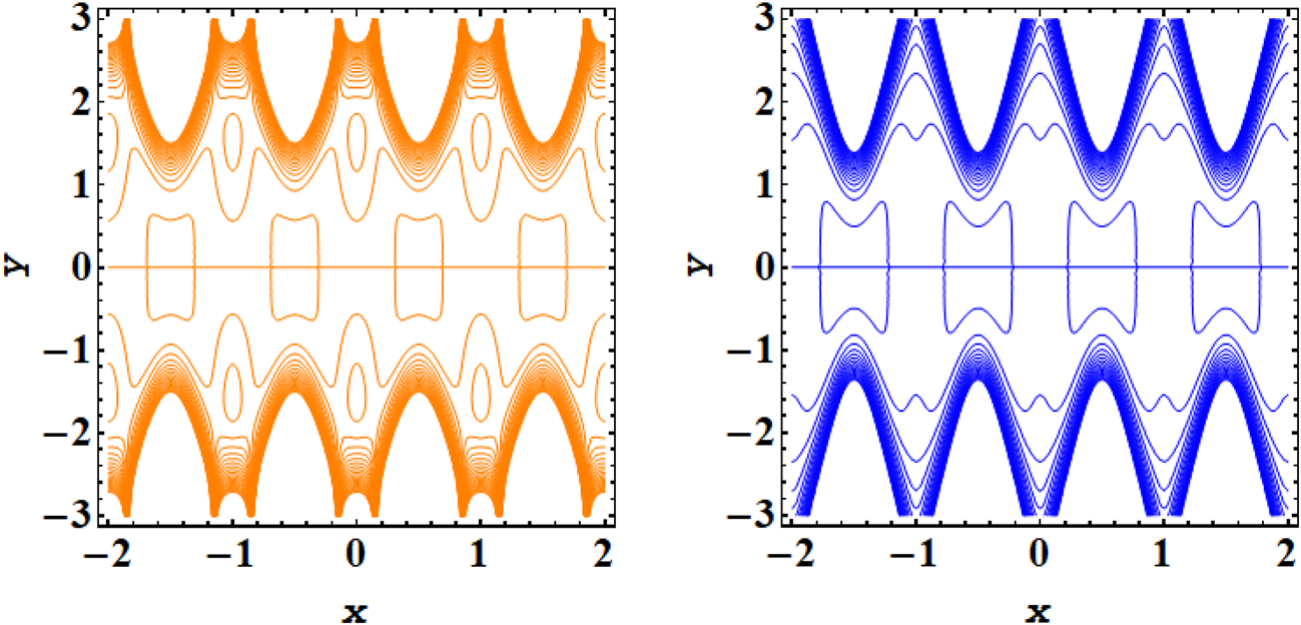
Streamlines outcomes for symmetric channel when ${U}_{HS}$= − 2, − 4.

**Figure 14:**
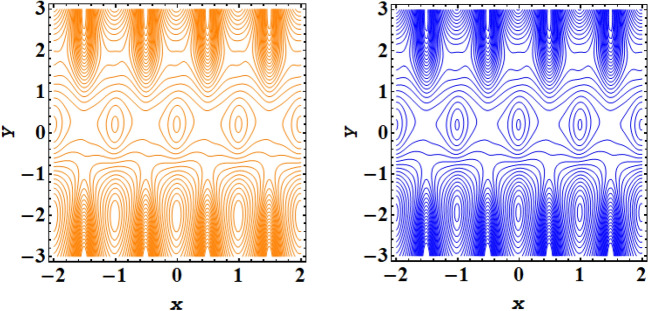
Streamlines outcomes for asymmetric channel when ${U}_{HS}$= − 2, − 4.

Streamline plot for symmetric conduit case with incrementing values of $\xi$ is presented in Fig. 11, while Fig. 12 presents the streamline plot for asymmetric conduit case with incrementing values of $\xi$. An exquisite flow pattern is revealed by these streamline graphs. These figures shows that the trapping size of trapping bolus increases with the rising values of couple stress fluid due to high shear rate of the fluid and for asymmetric channel case more streamlines are going to enclose and no of trapping bolus increases with the rise in ξ. Further for symmetric conduit case behavior of streamlines is same in the upper and lower part of the channel, However in asymmetric case behavior of streamlines is different, as in this case more contours are going to be enclose. In case of asymmetric conduit, the incrementing values of $\zeta$ results in decline of trapping magnitude. If we compare the streamline pattern for symmetric in addition to asymmetric case, then an oval trapping configuration is observed in central region for symmetric case. Streamline plot for symmetric conduit case with incrementing values of Helmholtz–Smoluchowski velocity ${U}_{HS}$ is presented in Fig. 13, while Fig. 14 presents the streamline plot for asymmetric conduit case with incrementing values of ${U}_{HS}$. These figures shows that the size of trapping bolus increases with the rising values of ${U}_{HS}$ as increase in ${U}_{HS}$ negative direction physically implies the presence of a strong electric field which produces an enhancement in the velocity profile and size of trapping bolus increases and for asymmetric channel case more streamlines are going to enclose and no of trapping bolus increases with the rise in ${U}_{HS}$. Further for symmetric conduit case behavior of streamlines is same in the upper and lower part of the channel, However in asymmetric case behavior of streamlines is different, as in this case more contours are going to be enclose. In case of asymmetric conduit, the incrementing values of ${U}_{HS}$ results in decline of trapping magnitude. Validity of results have been done by comparing the currents results with existing literature in Table 1 and Table 2.

**Table 1:** Comparison of present results with the existing literature for symmetric channel case.

y	u(x,y) when ${U}_{HS}=0,$ and no cilia case	u(x,y) for Ref.^25^ when M = 0	u(x,y) for Ref.^34^ when M = 0
0.0	$-0.997316$	$-0.997326$	$-0.997326$
0.2	$-0.997410$	$-0.997450$	$-0.997450$
0.4	$-0.997679$	$-0.997669$	$-0.997669$
0.6	$-0.998117$	$-0.998127$	$-0.998127$
0.8	$-0.998839$	$-0.998849$	$-0.998849$
1	− 1.000000	− 1.000000	− 1.000000

**Table 2:** Comparison of present results with the existing literature for asymmetric channel case.

y	u(x,y) when $${U}_{HS}=0, $$and no cilia case	u(x,y) for Ref.^39^ when M = 0
-1.0	$-1.00000$	$-1.00000$
-0.7	$-0.997410$	$-0.997450$
-0.3	$-0.997679$	$-0.997669$
-0.2	$-0.998117$	$-0.998127$
0.4	$-0.997839$	$-0.998849$
0.6	$-0.997679$	$-0.997579$
0.8	$-0.997410$	$-0.997810$
1.0	− 1.000000	− 1.000000

## Conclusion

This mathematical model encloses the comparison analysis of Couple stress fluid in a symmetric in addition to asymmetric conduit. Major highlights of current analysis are given as

1. The flow profile has higher values for asymmetric conduit in relation to the symmetric conduit.
2. The numerical values of $\Delta P$ are higher for an asymmetric conduit in comparison to the symmetric conduit.
3. As the flow tends to a Newtonian profile, a declining magnitude of bolus is observed.
4. Pressure gradient has maximum numerical values in central region of conduit while it diminishes towards the boundaries.
5. Helmholtz–Smoluchowski velocity ${U}_{HS}$ rises pressure rise for both symmetric and asymmetric conduit case respectively.
6. Electromagnetic radiation generated by the thermal motion of particles in matter decreases temperature profile.
7. It is observed that the temperature profile decreases with the rise in Prandtl number.
8. An integrated analysis on Metachronal propulsion plus peristaltic flow is highlighted in detail.
9. The size of trapping bolus increases with the rising values of ${U}_{HS}$ due to strong electric field**.**
10. In asymmetric case behavior of streamlines is different, as in this case more contours are going to be enclosed.

## Data Availability

The datasets used and/or analyzed during the current study available from the corresponding author on reasonable request.

## List of symbols

$\left(X,Y\right)$: Cartesian system of coordinates
$\alpha$: Eccentricity for elliptical movement
$c$: Speed of wave propagation
$$Re$$: Reynold number
${d}_{ij}$: Velocity gradient (symmetric part)
${\tau }_{ij}$: Stress tensor (symmetric part)
$${T}_{ij}, {M}_{ij}$$: Stress tensor
$${w}_{i}$$: Vorticity vector
$$\beta $$: Wave number
$\zeta$: Couple stress parameter
${\rho }_{e}$: Local electric charge density
$$k$$: Thermal conductivity
$Q$: Dimensionless flow rate
$$\left(u,v\right)$$: Velocity components
$\varepsilon$: Parameter of cilia length
$$t$$: Time
$$\lambda $$: Wavelength
$$P$$: Pressure
$${v}_{i}$$: Velocity vector
${T}_{ij}^{A}$: Stress tensor (asymmetric part)
$${\delta }_{ij}$$: Kronecker delta
$$a$$: Conduit radius
$$\rho $$: Density
${\text{E}}_{{{{\bar{\text{x}}}}}}$: The axial electric field
$${q}_{r}$$: Radiation factor
$$\Delta P$$: Pressure rise
